# Clinical Utility of Magnetic Resonance Urethrography in Assessment of Canine Urethra

**DOI:** 10.1111/jvim.70236

**Published:** 2025-09-17

**Authors:** Younjin Kang, Jonghui Baek, Sunghwa Hong, Soyeon Kim, Eunjee Kim, Junghee Yoon, Jihye Choi

**Affiliations:** ^1^ College of Veterinary Medicine Seoul National University Seoul Korea

**Keywords:** computed tomography, lower urinary tract, magnetic resonance imaging, transitional cell carcinoma, uroepithelial tumor

## Abstract

**Background:**

Magnetic resonance imaging (MRI) has been widely used in human medicine for evaluating lower urinary tract diseases; however, its application in veterinary medicine remains limited.

**Hypothesis/Objectives:**

To compare MRI with computed tomography (CT) in visualizing urethral and bladder wall layers and to assess the feasibility and diagnostic utility of MRI for evaluating lower urinary tract anatomy and pathology in dogs. It was hypothesized that the T2‐weighted (T2W) sequence would provide the most distinct visualization of urethral layers, while the contrast‐enhanced T1‐weighted (post‐T1W) sequence would best delineate bladder wall layers.

**Animals:**

Five healthy dogs and six dogs with suspected uroepithelial tumors.

**Methods:**

Magnetic resonance imaging sequences (T2W, pre‐T1W, and post‐T1W) and contrast‐enhanced CT (post‐CT) were performed. Images were evaluated for urethral and bladder wall layer distinction, urethral conspicuity, image quality, and characteristics of lower urinary tract tumors.

**Results:**

T2W sequence clearly delineated urethral wall layers and differentiated the urethral mucosal layer from the lumen using urine as a natural contrast. Post‐T1W sequence enhanced bladder wall layer visualization, improving anatomical boundary conspicuity and aiding in tumor invasion detection. In contrast, post‐CT allowed rapid imaging with minimal motion artifacts but provided limited soft‐tissue detail.

**Conclusion and Clinical Importance:**

Magnetic resonance imaging demonstrated superior efficacy in evaluating anatomic structures and lesions of the lower urinary tract, particularly in assessing tissue invasion. Combining T2W and post‐T1W sequences optimized diagnostic accuracy, making MRI a valuable tool for assessing lower urinary tract pathology in dogs.

**Abbreviations**
ADC: apparent diffusion coefficient
BTA: bladder tumor antigen
CT: computed tomography
DCE: dynamic contrast‐enhanced
DWI: diffusion‐weighted imaging
FOV: field of view
FS: fat‐saturated
HU: Hounsfield units
IACUC: Institutional Animal Care and Use Committee
ICC: intraclass correlation coefficient
IV: intravenous
MIBC: muscle‐invasive bladder cancer
MR: magnetic resonance
MRI: magnetic resonance imaging
NEX: number of excitations
PACS: picture archiving and communication system
post‐CT: post‐contrast computed tomography
post‐T1W: contrast‐enhanced T1‐weighted
pre‐T1W: pre‐contrast T1‐weighted
SNR: signal‐to‐noise ratio
SNU: Seoul National University
SPSS: Statistical Package for the Social Sciences
T1W: T1‐weighted
T2W: T2‐weighted
TCC: transitional cell carcinoma
TE: echo time
TR: repetition time
VI‐RADS: Vesical Imaging‐Reporting and Data System

## 1 Introduction

Lower urinary tract tumors account for approximately 2% of all canine malignancies, with many affecting both the bladder and urethra [1, 2, 3, 4]. The involvement of the urethra has clinical implications, as it significantly limits complete surgical excision and is associated with higher metastasis rates and a poorer prognosis compared to tumors confined to the bladder [3, 4, 5]. Urethral invasion can complicate interventional procedures including endoscopic or fluoroscopically guided stent placement due to luminal narrowing and risks of migration [6], while radiation may cause late complications such as urethral stricture [7]. Accurate assessment of the urethra is therefore essential for proper treatment planning and prognosis.

The canine urethra comprises three histologically layers—mucosa, submucosa, and muscle—with gender‐based anatomical differences [8, 9]. Among imaging modalities, retrograde urethrography is primarily used to assess the urethral lumen [10]. While it is inexpensive and easily accessible, its invasive nature, potential complications such as urethral trauma or infection, and inability to assess surrounding soft tissues require complementary imaging techniques [11, 12]. Ultrasonography is widely used due to its accessibility and ability to assess the urethral wall and lumen without anesthesia [13]. Nevertheless, it has limitations in visualizing the lumen‐mucosal lining in the collapsed urethra [14, 15]. Sono‐urethrography using saline, agitated saline, and a contrast agent has been explored to overcome these limitations, but ultrasound still allows only partial visualization of the urethra due to pelvic bone shadowing [15].

Computed tomography (CT) and magnetic resonance imaging (MRI) are increasingly used to delineate tumors in the lower urinary tract. CT offers detailed three‐dimensional images, but its soft‐tissue contrast is suboptimal [16]. In contrast, MRI offers superior soft‐tissue resolution and has become a valuable tool in human medicine for evaluating urethral tumors [17, 18, 19]. MR urethrography detected findings such as partial urethral stricture, periurethral fibrosis, and prostatic displacement that were undetected by conventional imaging in human patients [11]. Similarly, in a case report of a dog with suspected transitional cell carcinoma (TCC), MRI—particularly T2‐weighted (T2W) and contrast‐enhanced T1‐weighted (post‐T1W) imaging—clearly demonstrated muscular disruption of the bladder and urethra, appearing to outperform CT in detecting tumor invasion [20]. In humans, MRI features of the normal urethra reflect the histological layers. On axial T2W and post‐T1W, the urethra displays a target‐like appearance: an outer hypointense ring corresponds to the striated muscle, a middle hyperintense ring represents the submucosa and smooth muscle, and an inner hypointense core reflects the mucosa [21]. These MRI features aid in detecting tumors and assessing local invasion [21]. Meanwhile, in veterinary medicine, the application of MRI to the canine urethra is limited, with only one low‐field study reporting limited ability to differentiate between smooth and striated muscle in normal dogs [22].

The purpose of this study was to evaluate the feasibility of MR urethrography for depicting both normal and abnormal conditions of the canine lower urinary tract, as well as to determine the most effective sequences for urethral evaluation. This study hypothesizes that MR urethrography will provide clearer images than CT and that the T2W sequence will effectively visualize the urethral layers.

## 2 Materials and Methods

This study was conducted with the approval of the Institutional Animal Care and Use Committee of Seoul National University, and all animals were maintained according to the Guidelines for Animal Experiments of Seoul National University (SNU IACUC‐24010‐3). Informed owner consent was obtained for all client‐owned patients prior to inclusion in the study.

### 2.1 Study 1. MR and CT Evaluation of the Normal Urinary Tract Group

#### 2.1.1 Animals

This prospective, method‐comparison study included four purpose‐bred Beagles (three castrated males, one intact male) and one client‐owned castrated male Labrador Retriever with a history of pelvic osteosarcoma and pelvectomy. The Labrador was included based on the absence of urinary abnormalities on physical examination, diagnostic imaging, and medical history. The median age of the dogs was 6 years (range: 6–8 years), and the median body weight was 15.5 kg (range: 12.7–35.6 kg). All Beagles were confirmed clinically healthy based on physical examination, blood tests, and thoracic and abdominal radiographs. None of the dogs had a history or signs of urinary diseases.

#### 2.1.2 Anesthesia and Schedule

All MRI and CT scans were performed under general anesthesia. After placing a 22–24 gauge intravenous catheter in the cephalic vein, anesthesia was induced with medetomidine (0.02 mg/kg IV, Domitor, Zoetis, Helsinki, Finland) and alfaxalone (2.0 mg/kg IV, Alfaxan, Jurox Pty Ltd., Rutherford, Australia). Anesthesia was maintained with sevoflurane (Sevofran, Hana Pharm Co. Ltd., Seoul, South Korea) in oxygen (1–1.2 L/min) via an endotracheal tube. Physiological parameters including heart rate, respiratory rate, blood pressure, oxygen saturation, and body temperature were monitored using the CARESCAPE Monitor B650 (GE Healthcare, Helsinki, Finland) for CT and the Datex‐Ohmeda Monitor, Type N‐MRI 2‐00 (GE Healthcare, Helsinki, Finland) for MRI. MRI was performed first, followed by CT.

#### 2.1.3 MRI Scan

The dog was positioned in sternal recumbency with hindlimbs extended cranially and feet‐first. Magnetic resonance imaging was performed using a 1.5 Tesla scanner (SIGNA CREATOR, GE Healthcare, Chicago, IL, USA) with an 8‐channel flex coil wrapped around the pelvis. Slice thickness was 3.0 mm in Beagles and 4.0 mm in the Labrador Retriever. Slice gaps were as follows: 0.3 mm for the transverse and dorsal planes and 0.6 mm for the sagittal plane in Beagles; 0.4 mm for the sagittal and dorsal planes and 0.8 mm for the transverse plane in the Labrador Retriever. No artificial distention or compression was applied to the bladder or urethra. Motion‐reduction techniques such as respiratory triggering or breath‐holding were not used. Field of view was adjusted from the bladder neck to the distal membranous urethra. After obtaining all three plane images with T2W sequences as localizers, MR images in the transverse, dorsal, and sagittal planes were acquired through T2W, pre‐contrast T1‐weighted (pre‐T1W), and post‐T1W sequences. A dose of 0.1 mmol/kg of a gadolinium‐based contrast agent (Dotarem, 0.5 mmol/mL; Guerbet SA, Villepinte, France) was intravenously administered, and post‐T1W images were immediately acquired in the transverse, dorsal, and sagittal planes. Scan parameters are presented in Table 1.

**TABLE 1:** Magnetic resonance imaging scanning parameters for evaluation of the lower urinary tract in healthy dogs by sequence and imaging plane.

Parameter	T2W			Pre‐T1W			Post‐T1W		
	Trans	Dor	Sag	Trans	Dor	Sag	Trans	Dor	Sag
TR (ms)	5390–9028	2861–5117	2563–4840	317–727	317–772	400–617	322–736	450–772	400–596
TE (ms)	90–122	94–114	87–106	9–15	9–10	8–11	9–15	10–11	8–11
NEX	3	1–3	3–4	1–3	2–4	2–4	1–3	2–4	2–4
FOV (mm)	140–260	160–290	180–220	140–260	160–290	180–220	140–280	160–290	180–220

Abbreviations: Dor, dorsal; FOV, field of view; NEX, number of excitations; post‐T1W, contrast‐enhanced T1‐weighted; pre‐T1W, T1‐weighted; Sag, sagittal; T2W, T2‐weighted; TE, echo time; TR, repetition time; Trans, transverse.

#### 2.1.4 CT Scan

Each dog was positioned in sternal recumbency with the extended hindlimbs and head‐first. CT scans were acquired using a 160 multi‐slice scanner (Aquilion Lightning, Canon Medical System, Tustin, CA, USA) with the following parameters: 120 kVp, 200 mA, 0.5 mm slice thickness, and 1.0 s rotation time and a spiral pitch of 0.81. The FOV included the entire abdomen and pelvis. Following a pre‐contrast CT scan, a test bolus scan was conducted at the level of the descending aorta and caudal vena cava after injection of 1 mL/kg of iohexol (Omnipaque 300, GE Healthcare, Oslo, Norway) using a power injector (Medrad Stellant, Bayer HealthCare, Whippany, NJ, USA) at a rate of 2.5 mL/s to determine the scan delay for arterial and venous imaging. A breath‐holding technique was used to minimize respiratory motion artifacts. Subsequently, arterial, venous, and delayed‐phase CT images were acquired with the administration of 2 mL/kg of iohexol at the same rate. The acquired image dataset was reconstructed in transverse, sagittal, and dorsal planes, at 0.5 mm slice thickness and interval.

After completion of CT and MRI, the general condition of the dogs was monitored for 5 days, along with any side effects related to anesthesia or contrast agent.

#### 2.1.5 Image Analyses

All transverse, sagittal, and dorsal planes of MRI and CT images were evaluated on a picture archiving and communication system (Infinitt PACS; INFINITT Healthcare, Seoul, South Korea) by two independent observers (Y.K. and J.B.), each with 2 years of radiology experience. The images were presented to the observers in random order. The observers were not completely blinded to the technique because of inherent differences in the image characteristics of the CT and MRI. The contrast‐enhanced CT (post‐CT) images were assessed based on the overall contrast enhancement by combining the arterial, venous, and delayed phases using a soft tissue window setting (window level: 40 Hounsfield Units [HU], window width: 400 HU). In MRI, T2W, pre‐T1W, and post‐T1W sequences were assessed individually.

For qualitative assessment, wall layer distinction, conspicuity of the urethra, and image quality were subjectively scored using a 4‐point scale (Table 2). The wall layer distinction was defined as the ability to identify the anatomically distinct layers relevant to each region, based on histological anatomy. In image interpretation, layers that showed different signal intensities were considered as separate anatomic layers, and the clarity of the margins between layers was assessed. The number and type of layers considered varied depending on anatomical sites due to differences in histological composition. Therefore, layer distinction was evaluated at three anatomical sites: (1) the prostatic urethra, (2) membranous urethra, and (3) bladder. The conspicuity of the urethra was assessed based on the visibility and sharpness of the boundaries: (1) between the prostatic urethra and prostatic parenchyma, (2) between the inner wall of the membranous urethra and its lumen, and (3) the outer wall of the membranous urethra with the rectum and surrounding muscles. Overall image quality was evaluated by assessing the presence and effect of artifacts on the clarity of the image.

**TABLE 2:** Evaluation criteria for the qualitative assessments of the lower urinary tract using CT and MRI.

Evaluation factor	Scoring criteria
Wall layer distinction	1. No distinction between the layers 2. Layers barely visible 3. Layers visible but slightly blurred 4. Layers clearly and distinctly visible
Conspicuity of the urethra	1. Not discernible 2. Minimally discernible 3. Discernible but slightly blurred 4. Clearly discernible structures
Image quality	1. Uninterpretable due to significant artifacts 2. Artifacts present, affecting image evaluation 3. Minimal artifacts with no impact on interpretation 4. Optimal image quality with no artifacts

### 2.2 Study 2. MR and CT Evaluation of the Uroepithelial Tumor Group

This retrospective study included six client‐owned dogs diagnosed with uroepithelial tumors between October 2023 and June 2024 at Seoul National University Veterinary Medical Teaching Hospital. The inclusion criteria of dogs were as follows: (1) the gross mass identified in the urethra, bladder, or prostate, (2) both CT and MR urethrography performed on the same day, and (3) a suspected or confirmed diagnosis of uroepithelial tumors based on one or more of the following: bladder tumor antigen (BTA) test, traumatic catheterization, B‐Raf proto‐oncogene, serine/threonine kinase (BRAF) testing, or biopsy. However, the histologic confirmation was not required for inclusion. Anesthesia and CT and MRI imaging protocols were consistent with normal groups. All images were evaluated by one observer (Y.K.).

### 2.3 Statistical Analyses

Statistical analyses for normal dogs were conducted by a statistician (E.K.) using SPSS Statistics (Version 27 for Windows, IBM Corp., Chicago, IL, USA). Data normality was assessed using the Shapiro–Wilk test. Differences among post‐CT, T2W, pre‐T1W, and post‐T1W for each evaluation factor were evaluated using the Kruskal–Wallis test, followed by post hoc comparisons with the Mann–Whitney *U*. A *p* value of < 0.05 was considered statistically significant. Interobserver agreement was assessed using the interclass correlation coefficient (ICC). ICC values below 0.5 were deemed poor, 0.5–0.749 fair, 0.75–0.9 good, and above 0.9 excellent [23].

## 3 Result

### 3.1 Study 1. MR and CT Evaluation of Normal Urinary Tract

Magnetic resonance imaging and CT evaluation were successfully completed without any side effects related to anesthesia, contrast agent, or imaging techniques in all dogs. Estimated acquisition times were derived from protocol parameters and typical sequence durations. The mean acquisition times were approximately 9, 5, and 3 min for transverse, dorsal, and sagittal plane acquisitions, respectively, for T2W sequences and 13, 7–9, and 3 min for transverse, dorsal, and sagittal acquisitions of pre‐ and post‐contrast T1W sequence. Pre‐ and post‐CT acquisitions required approximately 16 min. ICC was good to excellent for all MRI and CT images (Table 3).

**TABLE 3:** Interclass correlation coefficient values for interobserver reliability in the qualitative assessments of MRI images and CT of the canine lower urinary tract.

Evaluation factors	Evaluation sites	MRI	CT
Wall layer distinction	Prostatic urethra	0.96	1.00
	Membranous urethra	0.88	0.95
	Bladder	1.00	1.00
Conspicuity of the urethra	Outer membranous urethra	0.85	1.00
	Inner membranous urethra	1.00	1.00
	Outer prostatic urethra	0.93	1.00
Image quality		0.93	0.75

**FIGURE 1:**
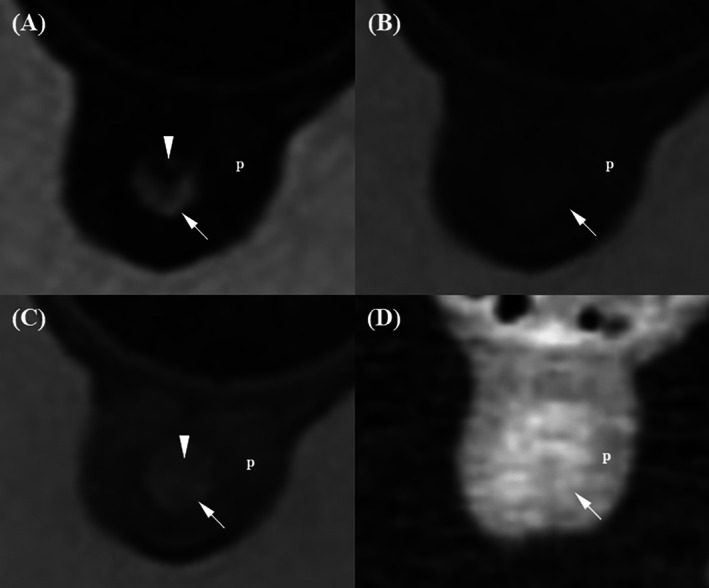
Magnetic resonance imaging and CT images of the prostatic urethra in a normal dog. (A) On the T2‐weighted image, the prostatic urethral wall appears as two distinguishable layers: a hyperintense outer layer (arrow) and a hypointense inner layer (arrowhead). The hyperintense outer layer of the prostatic urethra was clearly distinguishable from the hypointense prostatic parenchyma (p). (B) On the pre‐contrast T1‐weighted image, the prostatic urethra (arrow) appears as a single layer, isointense relative to the prostatic parenchyma (p). (C) On the contrast‐enhanced T1‐weighted image, the prostatic urethra appears as two blurred layers, with the outer layer (arrow) appearing brighter than the hypointense inner layer (arrowhead). The outer layer enhances more strongly than the surrounding prostatic parenchyma (p). (D) On the contrast‐enhanced CT image, the urethral wall appears as a single layer (arrow), slightly more enhanced than the prostatic parenchyma (p).

The evaluation data are presented in Table 4. In the prostatic urethra, its wall was visualized as two slightly blurred layers on T2W and post‐T1W images (Figure 1). T2W images showed the highest prostatic urethral layer distinction score, showing a hyperintense outer layer and a hypointense inner layer. Then, post‐T1W showed an outer layer with brighter enhancement than the inner layer (*p* = 0.02). In contrast, both pre‐T1W and post‐CT showed the prostatic urethra wall as a single, undifferentiated layer and showed a significantly lower layer distinction score compared to T2W and post‐T1W. In addition, T2W received the highest scores for the conspicuity of the prostatic urethral wall relative to prostate parenchyma, clearly delineating the hyperintense urethral wall against the hypointense adjacent prostate. Although post‐T1W images had significantly higher conspicuity scores than pre‐T1W and post‐CT, the urethral wall appeared discernible but slightly blurred. On pre‐T1W and post‐CT images, the urethral wall and prostate parenchyma showed similar brightness and no significant difference between the two modalities (*p* = 0.126).

**TABLE 4:** Qualitative assessments of the lower urinary tract using MRI and CT in healthy dogs.

Evaluation factors	Evaluation sites	MRI		CT	
		T2W	Pre‐T1W	Post‐T1W	Post‐CT
Wall layer distinction	Prostatic urethra	3.4 ± 0.70^a^	1.1 ± 0.32^b^	2.7 ± 0.48^c^	1.2 ± 0.42^b^
	Membranous urethra	4.0 ± 0.00^a^	2.5 ± 0.53^b^	3.7 ± 0.48^a^	2.0 ± 1.16^b^
	Bladder	1.00 ± 0.00^a^	2.40 ± 0.84^b^	3.00 ± 0.00^c^	1.00 ± 0.00^a^
Conspicuity of the urethra	Outer membranous urethra	2.30 ± 0.68^a^	2.30 ± 0.68^a^	3.00 ± 0.00^b^	1.0 ± 0.00^c^
	Inner membranous urethra	2.20 ± 1.03^a^	1.0 ± 0.00^b^	1.00 ± 0.00^b^	1.00 ± 0.00^b^
	Outer prostatic urethra	3.70 ± 0.48^a^	1.30 ± 0.48^b^	2.80 ± 0.63^c^	2.00 ± 1.16^b^
Image quality		2.90 ± 0.32^a^	2.20 ± 0.42^b^	2.20 ± 0.42^b^	3.70 ± 0.48^c^

*Note:* Data were presented as mean ± standard deviation. ^a–d^Letters assigned differently within a row indicate a significant difference according to the Mann–Whitney *U* test. The Kruskal–Wallis test showed significant differences among techniques for all evaluation factors (*p* < 0.001).

**TABLE 5:** Key imaging features of MRI and CT in clinical cases.

Case	Signalment	Tumor location	MRI findings	CT findings	MRI advantages over CT
1	11Y, SP Poodle	Entire urethra, extending to bladder neck	– T2W: bladder neck tumor sharply outlined by hyperintense urine – Post‐T1W: distinct “target‐like” appearance of urethral layers	Indistinct urethral wall layer	Superior urethral wall layer and tumor margin visualization
2	11Y, CM Poodle	Prostate, urethra, and bladder neck	– T2W and post‐T1W: clear delineation of the prostate‐rectal muscular layer – Post‐T1W: discernible urethra‐prostate margin with irregular contour	Indistinct prostate‐rectal muscular layer	Improved tumor margin delineation and exclusion of rectal invasion
3	12Y, CM Miniature Pinscher	Prostate, urethra, and bladder neck	– T2W and post‐T1W: clear delineation of the prostate‐rectal muscular layer	Indistinct prostate‐rectal muscular layer	Assisted in ruling out rectal invasion
4	9Y, SF Maltese	Bladder	– T2W: bladder tumor sharply outlined by hyperintense urine; focal bladder wall layer disruption	Tumor margin blurred by blooming artifact	Superior tumor margin visualization
5	14Y, CM Maltese	Bladder, prostate, and urethra	– T2W: bladder tumor sharply outlined by hyperintense urine; focal bladder wall layer disruption – Post‐T1W: partial distinction of inner and outer wall layers with disruption	Indistinct bladder wall layer	Superior bladder wall layer visualization
6	12Y, CM Maltese	Bladder, prostate, and urethra	– T2W: circumferential thickening of bladder with distinct wall layer and disrupted inner urethral layer–lumen interface	Indistinct bladder wall layer and inner urethral layer–lumen interface	Superior tumor invasion detection

Abbreviations: CM, castrated male, T2W, T2‐weighted; post‐T1W, contrast enhanced T1‐weighted; SP, spayed female.

**FIGURE 2:**
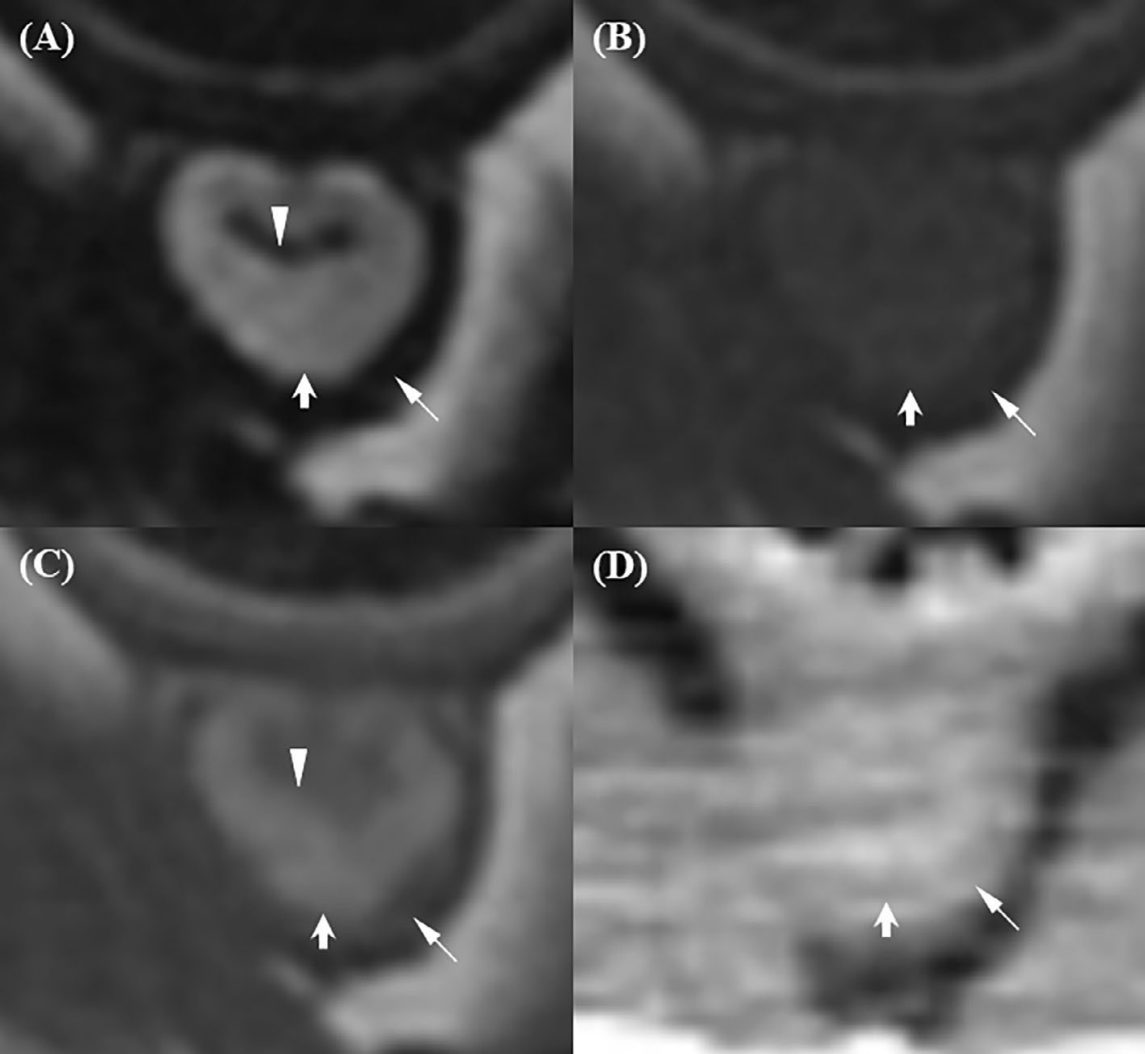
Magnetic resonance imaging and CT images of the membranous urethra in a normal dog. (A) On the T2‐weighted image, a target‐like appearance with three distinct layers is observed: hypointense (outer, long arrow), hyperintense (middle, short arrow), and hypointense (inner, arrowhead). (B) On the pre‐contrast T1‐weighted image, urethral layers are barely visible, with a slightly hyperintense inner layer (short arrow) and a hypointense outer layer (long arrow). (C) On the contrast‐enhanced T1‐weighted image, a target‐like appearance is observed: hypointense (outer, long arrow), hyperintense (middle, short arrow), and hypointense (inner, arrowhead), with the middle layer appearing brighter than the others. (D) On the contrast‐enhanced CT image, urethral layers are barely visible, with a strongly enhanced middle region (short arrow) and a less enhanced outer layer (arrow).

In the membranous urethra, a target‐like appearance with three distinct layers—hypointense, hyperintense, and hypointense from outer to inner—was observed on transverse planes of both T2W and post‐T1W images (Figure 2). In contrast, urethral layers were barely visible on pre‐T1W and post‐CT images. T2W and post‐T1W had significantly higher scores for wall layer distinction than pre‐T1W and post‐CT, with no statistically significant difference between the two (*p* = 0.067). The difference between pre‐T1W and post‐CT was not statistically significant (*p* = 0.231).

**FIGURE 3:**
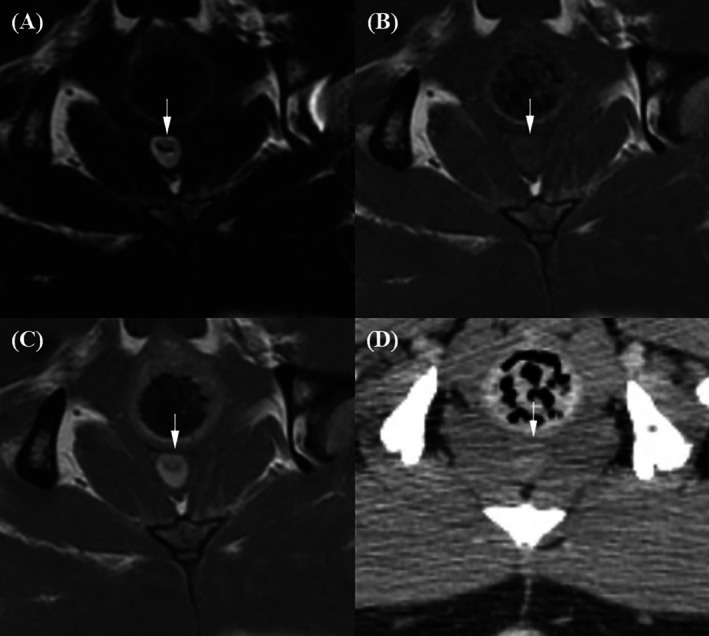
Magnetic resonance imaging and CT evaluation of the conspicuity of the outer membranous urethral layer (arrow) and the obturator internus muscle. (A) On the T2‐weighted image, the conspicuity between the outer urethral layer and the obturator internus muscle is minimally discernible. (B) On the pre‐contrast T1‐weighted image, the conspicuity remains minimally discernible. (C) On the contrast‐enhanced T1‐weighted image, the conspicuity is discernible but slightly blurred. (D) On the contrast‐enhanced CT image, the conspicuity is not discernible.

The membranous urethra is surrounded by pelvic muscles, particularly the obturator internus muscle, which remained in close contact caudally and affected urethral conspicuity on transverse images (Figure 3). In sagittal images, the membranous urethra was frequently overlapped by the rectum, making it difficult to distinguish the outer urethral layer from the adjacent rectal muscle. The outer layer of the membranous urethra appeared discernible but slightly blurred on post‐T1W images, which had the highest conspicuity scores among all techniques. Conspicuity was minimally discernible on T2W and pre‐T1W images, with no significant difference between them (*p* = 1.00). Post‐CT received the lowest possible scores, which were significantly lower than all MRI sequences.

**FIGURE 4:**
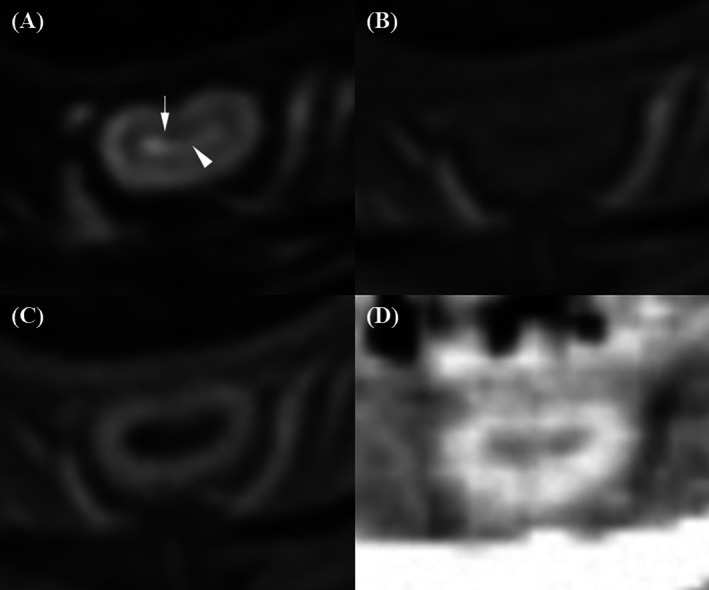
Magnetic resonance imaging and CT evaluation of the inner membranous urethral layer and lumen conspicuity. (A) On the T2‐weighted image, the conspicuity between the inner membranous urethral layer (arrow) and the lumen (arrowhead) is minimally discernible when the urethra is filled with urine. The urine in the lumen appears hyperintense, contrasting with the hypointense inner urethral layer. (B, C) On the pre‐contrast and contrast‐enhanced T1‐weighted images, respectively, the conspicuity is not discernible, with both the lumen and inner urethral layer appearing hypointense. (D) On the contrast‐enhanced CT image, the conspicuity is also not discernible. The lumen and inner urethral layer appear isoattenuating, surrounded by a strongly enhanced middle layer.

The inner layer of the membranous urethra and lumen were distinguishable only when the lumen was filled with urine in T2W, which appeared hyperintense and contrasted with the hypointense inner urethral layer (Figure 4). In contrast, on pre‐T1W and post‐T1W, the lumen appeared hypointense, with the inner urethral layer‐lumen indistinguishable. On CT images, the lumen was hypoattenuating compared to the strongly enhanced surrounding middle layer, making the inner urethral layer‐lumen conspicuity indistinct. Additionally, the strongly enhanced middle layer sometimes obscured lumen visualization due to blooming artifacts and partial volume effects on transverse images. T2W images received the significantly highest scores for inner urethral layer‐lumen conspicuity compared to all other techniques. Pre‐T1W, post‐T1W, and post‐CT images all received the lowest possible scores, as the inner urethral layer‐lumen interface was not discernible regardless of whether the urethra was filled with urine.

**FIGURE 5:**
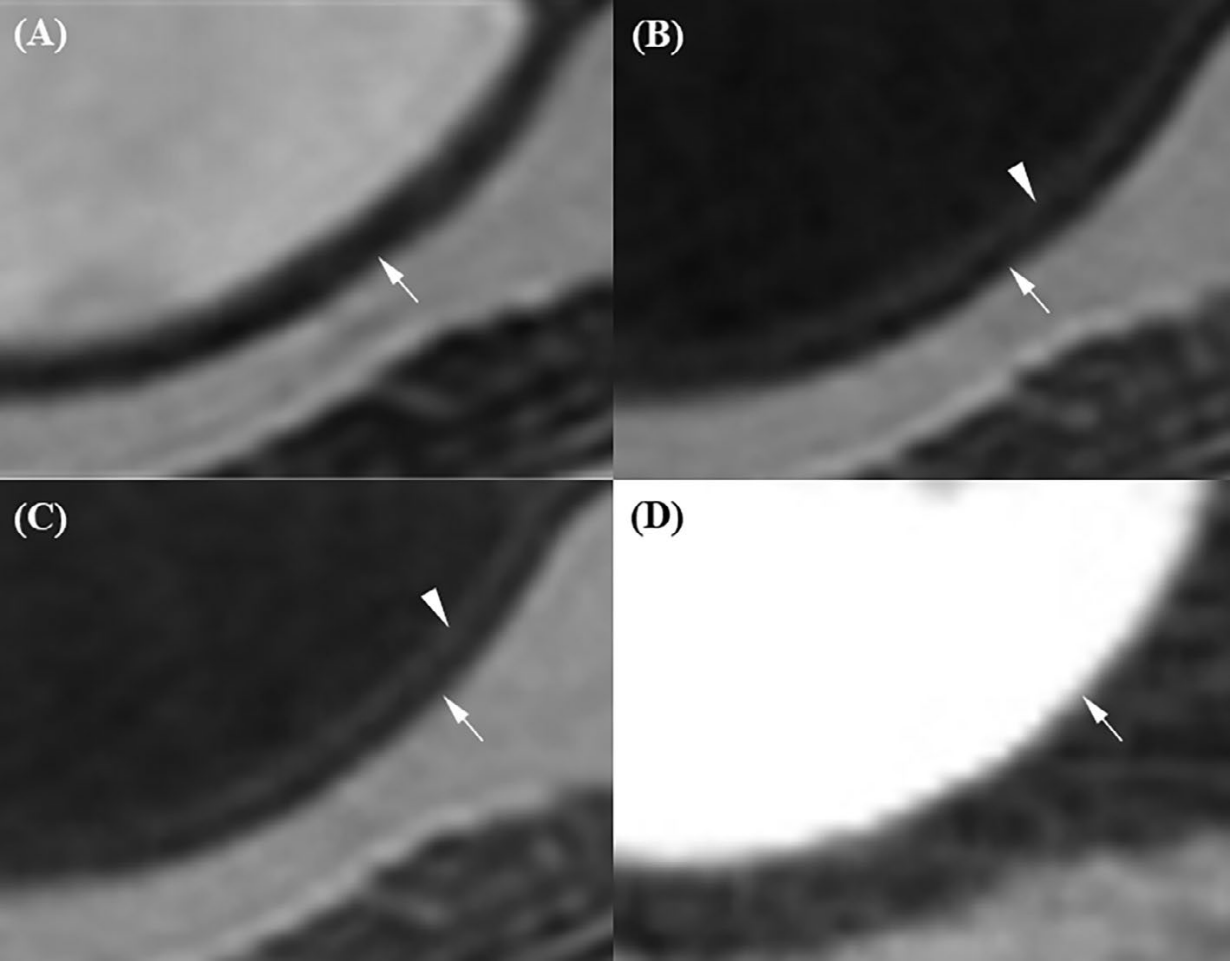
Magnetic resonance imaging and CT images of the bladder in a normal dog. (A) On the T2‐weighted image, the bladder wall appears as a single hypointense layer (arrow). (B) On the pre‐contrast T1‐weighted image, the bladder exhibits two barely discernible layers: a hyperintense inner layer (arrowhead) and a hypointense outer layer (arrow). (C) On the contrast‐enhanced T1‐weighted image, two bladder wall layers are visible but appear slightly blurred, with the inner layer (arrowhead) brighter than the outer layer (arrow). (D) On the contrast‐enhanced CT image, the bladder wall appears as a single layer (arrow) with mild enhancement.

In the urinary bladder, two layers consisting of a hyperintense inner layer and a hypointense outer layer were observed on both pre‐T1W and post‐T1W images (Figure 5). The ventral portion of the bladder on transverse pre‐ and post‐T1W images was the most consistently distinguishable region, while other areas—including those on dorsal sagittal planes—appeared mostly blurred. On T2W images, the bladder wall appeared as a single hypointense layer. In post‐CT images, the bladder also appeared as a single layer with mild enhancement. Statistical analysis demonstrated that post‐T1W images had the highest bladder wall layer distinction scores, followed by pre‐T1W (*p* = 0.03), while T2W and post‐CT received the lowest scores.

For the assessment of image quality and artifacts, motion artifact was the most prominent finding across all modalities. In MRI, additional random signal dropouts were frequently observed, particularly in pre‐T1W and post‐T1W sequences, most commonly in the anterior or posterior regions, extending beyond the range of the flex coil. Post‐CT had the highest image quality scores with minimal artifacts, followed by T2W (*p* = 0.001). Pre‐ and post‐T1W received the lowest scores, as artifacts impacted image interpretation, without a significant difference between the two (*p* = 1.00).

### 3.2 Study 2. MR and CT Evaluation of the Uroepithelial Tumor

The median age of the six dogs was 11.5 years (range: 9–14 years), and the median body weight was 5.8 kg (range: 3.0–8.0 kg). Uroepithelial tumors were diagnosed via BRAF testing or biopsy combined with traumatic catheterization in three dogs, traumatic catheterization alone in two dogs, and the BTA test alone in one dog. Clinical signs included hematuria, dysuria, polyuria, and dyschezia.

Two dogs had bladder masses invading the prostate and urethra, two had prostatic masses invading the urethra and bladder neck, one had a bladder mass without urethral involvement, and one had a urethral mass invading into the bladder (Table 5). Accordingly, the bladder in all six dogs was affected, the urethra was affected in five dogs, and the prostate was affected in four dogs.

**FIGURE 6:**
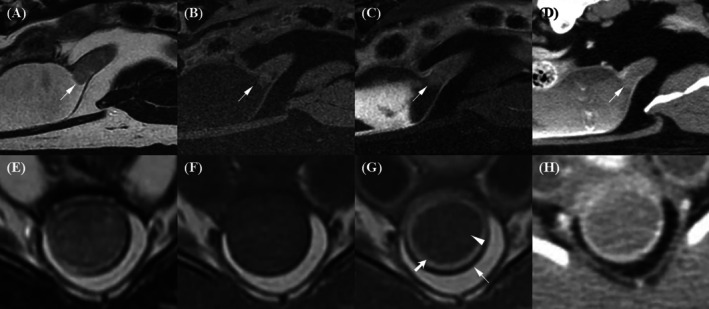
Magnetic resonance imaging and CT images of an 11‐year‐old spayed female Poodle with urethral transitional cell carcinoma (TCC). (A–D) sagittal and (E–H) transverse plane images: (A, E) T2‐weighted (T2W); (B) pre‐contrast T1‐weighted fat‐saturated (pre‐T1W FS); (C) contrast‐enhanced T1‐weighted fat‐saturated (post‐T1W FS); (D, H) contrast‐enhanced CT (post‐CT, venous phase); (F) pre‐contrast T1‐weighted (pre‐T1W); (G) contrast‐enhanced T1‐weighted (post‐T1W). (A) On the T2W, the cranial margin of the urethral tumor (arrow) is most clearly delineated, with the tumor sharply defined against the hyperintense urine. (E, F, H) On the T2W, pre‐T1W, and post‐CT images, respectively, the membranous urethral layers are barely visible. (G) On the post‐T1W, the urethra displays a distinct three‐layered “target‐like” appearance, consisting of an inner hypointense layer (arrowhead), a middle hyperintense layer (short arrow), and an outer hypointense layer (long arrow). The inner layer is abnormally thickened, while the middle layer is thinned.

A urethral tumor in an 11‐year‐old spayed female Poodle (Case 1) occupied the entire urethra, causing marked urethral dilation and extending into the urinary bladder (Figure 6). The bladder extension was consistently identified at the bladder neck on all imaging techniques and was most clearly delineated on T2W images, where the tumor was sharply outlined against the hyperintense urine. The lesion showed mild, homogeneous enhancement in both post‐T1W and post‐CT images. On transverse post‐T1W images, the tumor displayed a distinct three‐layered target‐like appearance, consisting of an abnormally thickened hypointense inner layer, a thinned hyperintense middle layer, and an outer hypointense layer. These layers were poorly visualized on T2W, pre‐T1W, and post‐CT images.

**FIGURE 7:**
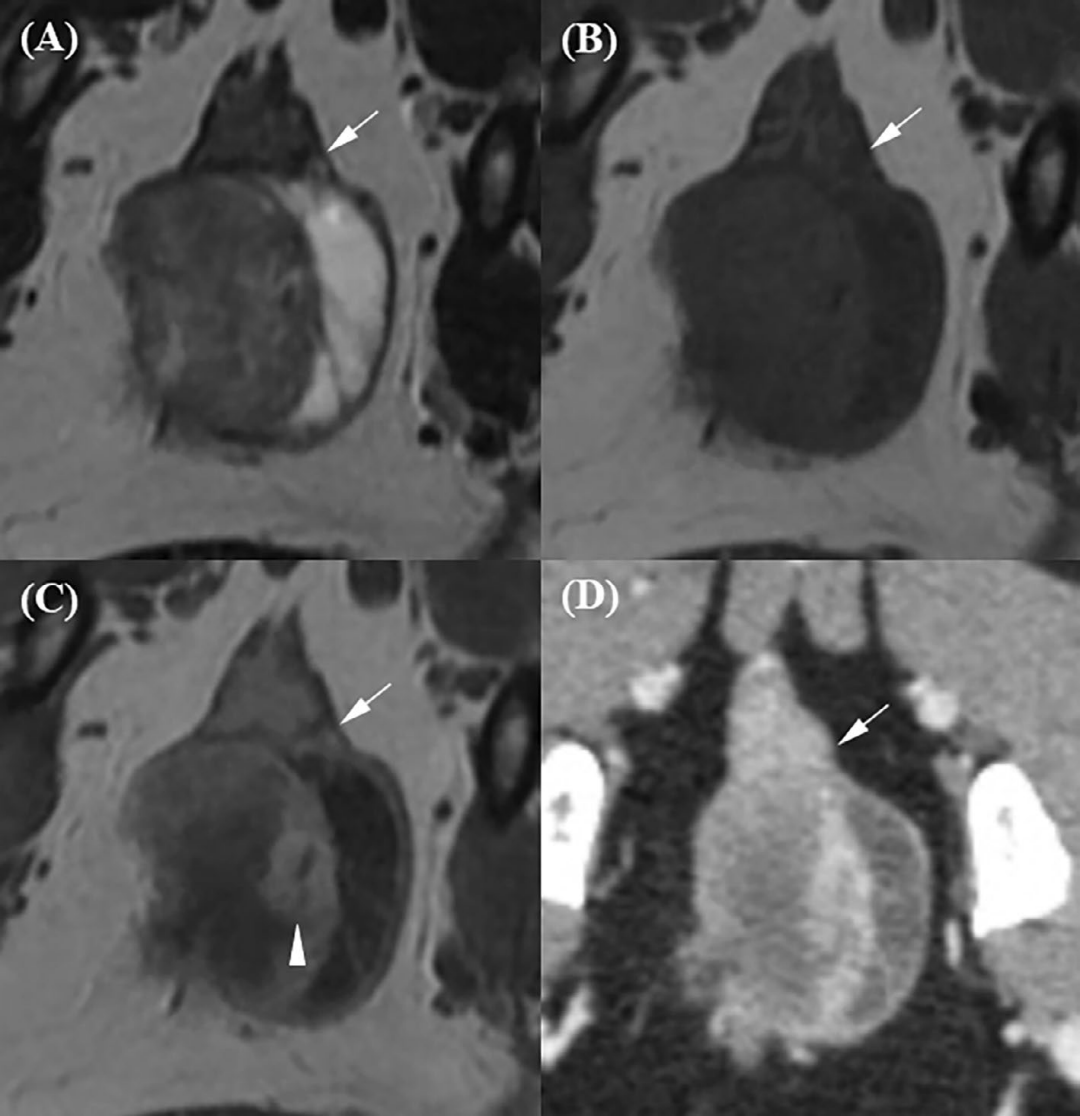
Magnetic resonance imaging and CT images of an 11‐year‐old castrated male Poodle with prostatic carcinoma. (A) On the T2‐weighted image, the urethra–prostate margin is not discernible, but the prostate–rectal muscular layer margin (arrow) is clearly discernible, with the hypointense rectal muscular layer remaining intact. (B) On the pre‐contrast T1‐weighted image, the urethra–prostate margin is not discernible, and the prostate–rectal muscular layer margin (arrow) is discernible but slightly blurred. (C) On the contrast‐enhanced T1‐weighted image, the urethra–prostate margin (arrowhead) is clearly discernible, revealing an irregular contour. The prostate–rectal muscular layer margin (arrow) is also clearly discernible, with the hypointense rectal muscular layer remaining intact. (D) On the contrast‐enhanced CT image, neither the urethra–prostate margin nor the prostate–rectal muscular layer margin (arrow) is clearly discernible.

**FIGURE 8:**
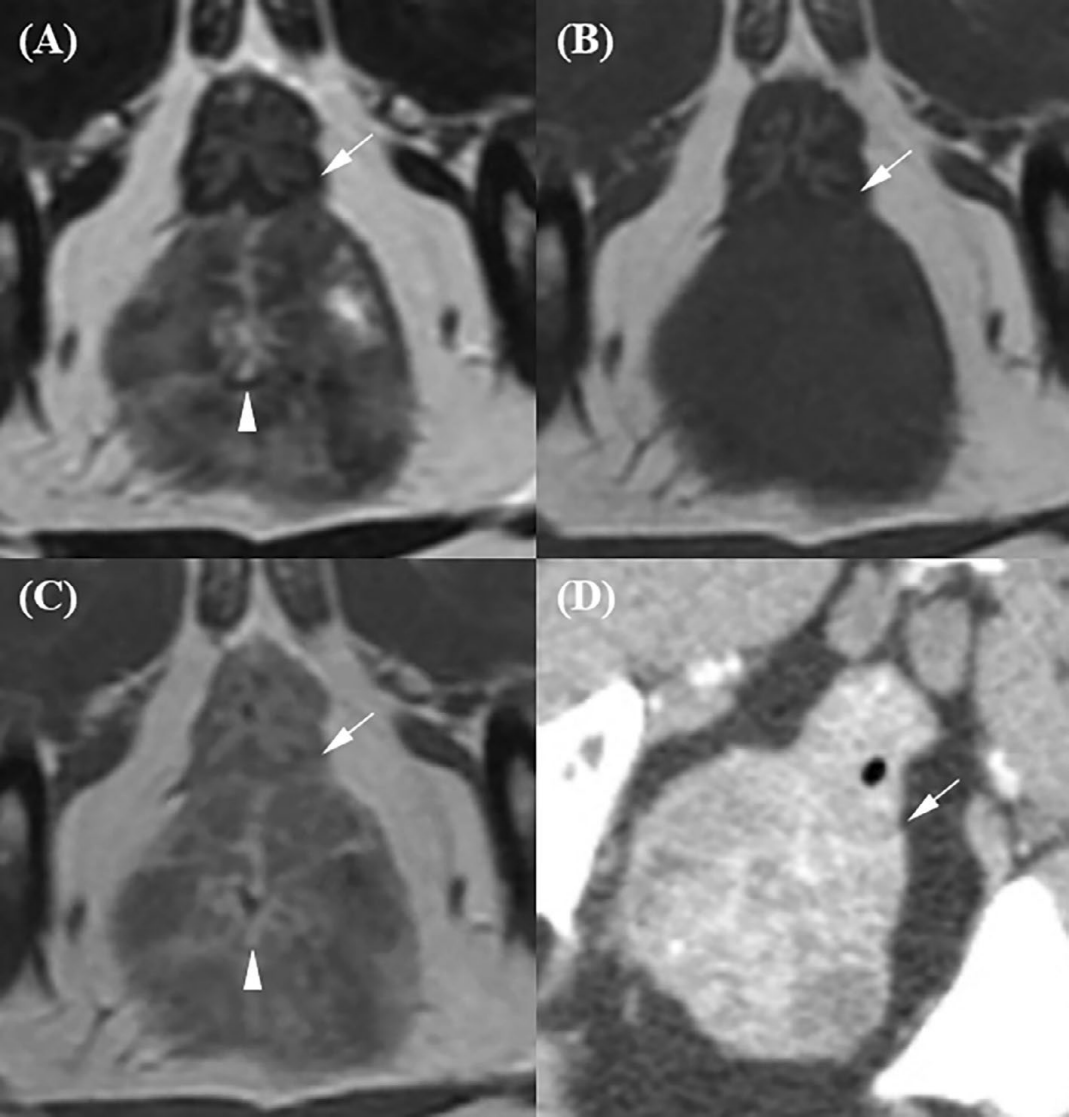
Magnetic resonance imaging and CT images of a 12‐year‐old castrated male Miniature Pinscher with prostatic carcinoma. (A) On the T2‐weighted image, the urethra–prostate margin (arrowhead) is discernible but slightly blurred. The prostate–rectal muscular layer margin (arrow) is clearly discernible, with the hypointense rectal muscular layer remaining intact. (B) On the pre‐contrast T1‐weighted image, the urethra–prostate margin is not discernible, while the prostate–rectal muscular layer margin (arrow) is discernible but slightly blurred. (C) On the contrast‐enhanced T1‐weighted image, the urethra–prostate margin (arrowhead) is discernible but slightly blurred. The prostate–rectal muscular layer margin (arrow) is clearly discernible, with the hypointense rectal muscular layer remaining intact. (D) On the contrast‐enhanced CT image, neither the urethra–prostate margin nor the prostate–rectal muscular layer margin (arrow) is discernible.

Prostatic tumors invading the bladder neck and urethra were found in an 11‐year‐old castrated male Poodle (Case 2) and a 12‐year‐old castrated male Miniature Pinscher (Case 3), accompanied by asymmetrical prostatic enlargement, irregular margins, cystic lesions, and mineralization (Figures 7 and 8). Compared to the surrounding pelvic and femoral muscles, the prostatic tumor exhibited heterogeneous mixed signals across all MRI sequences, with strong parenchymal enhancement. The margin between the prostatic urethra and prostate was not clearly discernible on all imaging modalities, except in Case 2, where post‐T1W distinctly revealed an irregular margin. Rectal compression was noted in both dogs. The margin between the prostate and rectum was clearly delineated on T2W and post‐T1W with preservation of the hypointense rectal muscle, mildly blurred on pre‐T1W, and not discernible on post‐CT images.

**FIGURE 9:**
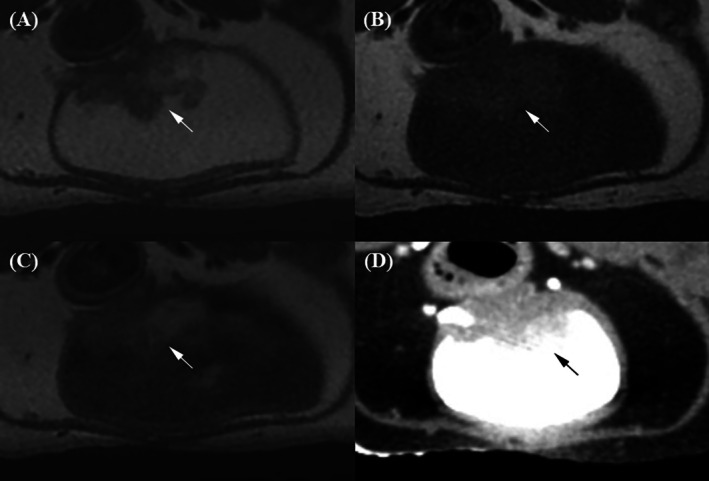
Magnetic resonance imaging and CT images of a 9‐year‐old castrated male Maltese with bladder transitional cell carcinoma (TCC). A broad‐based, irregular exophytic bladder tumor (arrow) was observed along the mid to caudal dorsal bladder wall. (A) On the T2‐weighted image, the bladder wall appears as a single hypointense, with evident disruption. The tumor margins are clearly delineated, contrasting with the hyperintense urine. (B, C) On the pre‐contrast T1W and contrast‐enhanced T1W images, respectively, the bladder wall layers and tumor margin are indistinct. (D) On the contrast‐enhanced CT image, the bladder wall appeared as a single isoattenuating layer, and the tumor margin is blurred due to blooming artifacts from the contrast medium.

**FIGURE 10:**
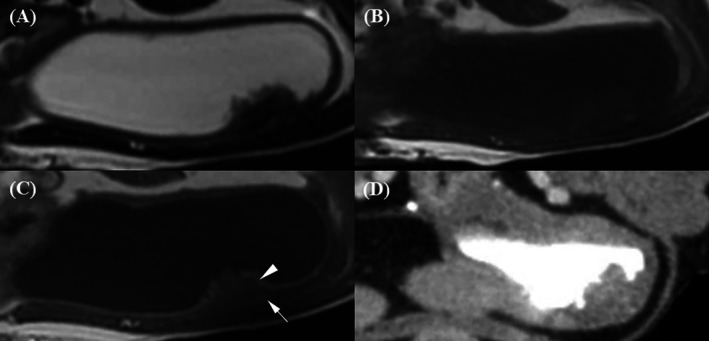
Magnetic resonance imaging and CT images of a 14‐year‐old castrated male Maltese with bladder transitional cell carcinoma (TCC). A broad‐based, irregular exophytic bladder tumor was observed along the cranioventral bladder wall. (A) On the T2‐weighted image, the bladder wall appears as a single hypointense layer with evident disruption. The tumor margins are clearly delineated, contrasting with the hyperintense urine. (B) On the pre‐contrast T1‐weighted image, the bladder wall layers and tumor margins are indistinct. (C) On the contrast‐enhanced T1‐weighted image, a clearly defined, strongly enhanced inner layer (arrowhead) and a less‐enhanced outer layer (arrow) are observed, with evident disruption due to tumor invasion. (D) On the contrast‐enhanced CT image, the bladder wall appears as a single isoattenuating layer, and the tumor margins are clearly delineated, contrasting with the contrast medium.

**FIGURE 11:**
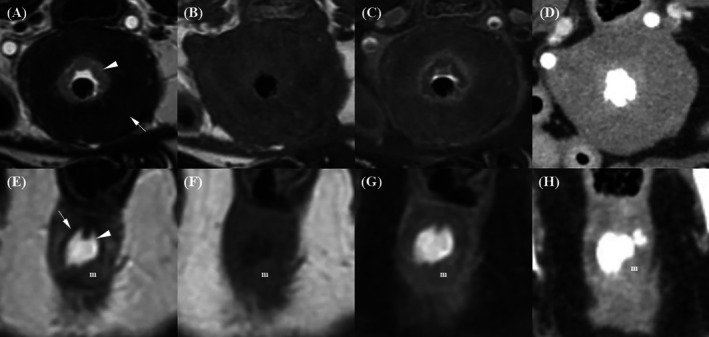
Magnetic resonance imaging and CT images of a 14‐year‐old castrated male Maltese with a suspected uroepithelial tumor invading the prostate. (A, E) T2‐weighted (T2W); (B, F) pre‐contrast T1‐weighted (pre‐T1W); (C, G) contrast‐enhanced T1‐weighted fat‐saturated (post‐T1W FS); and (D, H) contrast‐enhanced CT (post‐CT, venous phase) images. A mass causes significant circumferential bladder wall thickening and luminal narrowing. (A) On the T2‐weighted image, thickening of both the hyperintense inner layer (arrowhead) and hypointense outer layer (arrow) is observed. (E) The urethral lumen, filled with hyperintense urine, provides clear conspicuity between the inner layer (arrow) and the lumen (arrowhead), except in the area where a mass (m) protruding ventrally from the prostatic urethra disrupts this inner urethral layer–lumen margin.

Bladder tumor showing a broad‐based, irregular exophytic mass involving the mid‐to‐caudal dorsal bladder wall was found in a 9‐year‐old spayed female Maltese (Case 4) (Figure 9). The bladder tumor appeared hyperintense relative to the surrounding pelvic and femoral muscles on T2W and T1W with strong contrast enhancement. On T2W, the bladder wall appeared as a single hypointense layer with focal disruption, and the tumor margins were clearly delineated against the hyperintense urine. In contrast, the bladder wall layers and the tumor margin were indistinct on pre‐ and post‐T1W images. On post‐CT images, the bladder wall appeared as a single isoattenuating layer, and the tumor margin was blurred due to blooming artifacts from the contrast medium. Bladder tumors invading the prostate and urethra were identified in two castrated male Maltese dogs, aged 14 and 12 years (Cases 5 and 6). Both masses exhibited broad‐based, irregular morphology with associated bladder wall thickening and urethral narrowing (Figures 10 and 11). On T2W, the bladder wall appeared as a single hypointense layer with focal disruption or as circumferential thickening of the inner hyperintense and outer hypointense layers. Tumor margins were clearly delineated against the hyperintense urine. On post‐T1W, partial distinction between an enhancing inner layer and less‐enhanced outer layer was observed, while pre‐T1W and post‐CT images failed to delineate the wall layers clearly. In Case 6, a mass protruding ventrally from the prostatic urethra disrupted the inner urethral layer–lumen interface.

## 4 Discussion

This study evaluated the MRI and CT in visualizing the lower urinary tract in both healthy dogs and those with uroepithelial tumors. MRI, particularly T2W and post‐T1W sequences, achieved significantly higher scores than CT in the assessment of wall layer distinction and tissue conspicuity. In contrast, CT received the highest scores for overall image quality compared to all MRI sequences. T2W provided the most distinct visualization of the prostatic urethral wall, while both T2W and post‐T1W sequences achieved the highest scores for membranous urethral layer distinction. For bladder wall evaluation, post‐T1W demonstrated the best layer differentiation.

T2W and post‐T1W sequences demonstrated a characteristic target‐like appearance in normal canine membranous urethras displaying three distinct layers, consistent with human findings [21]: an outer hypointense layer that may correspond to striated muscle, a middle hyperintense layer that may represent submucosa and smooth muscle, and an inner hypointense layer that may reflect the mucosa [19, 21].

The prostatic urethra showed only two layers due to the lack of continuous circular smooth and striated muscle [9]. In dogs with urethral and prostatic tumors (Cases 1, 2, and 3), T2W and post‐T1W images consistently demonstrated disruption of the normal target‐like appearance of the urethra, suggestive of tumor invasion, and revealed an abnormally thickened urethral wall layer. In particular, post‐T1W images provided superior visualization of urethral wall layers, with clearer layer distinction and more conspicuous irregularities along tumor margins. The irregular margin between the urethra and prostate was more clearly identified on post‐T1W images than on T2W images. These findings emphasize the diagnostic value of contrast‐enhanced MRI in detecting subtle disruptions of urethral architecture associated with tumor invasion.

Similar to human medicine, where MRI plays a critical role in the local staging of urethral tumors and is superior to CT in detecting urethral recurrences post cystectomy for infiltrative carcinoma, our study supports the utility of T2W imaging for anatomical assessment and post‐T1W imaging for improved tumor conspicuity [19, 24, 25]. In human medicine, disruption of the target‐like urethral appearance is associated with periurethral invasion and correlates with clinically significant staging criteria [19]. These results suggest that MRI, particularly post‐T1W imaging, can determine the urethral tumor invasion and extent in canine urethral tumors.

The bladder wall in both dogs and humans consists of four histological layers, consisting of mucosa, submucosa, muscularis propria, and serosa or adventitia [26, 27, 28]. The outermost layer is typically not visualized on MRI in human medicine [28]. In healthy dogs, post‐T1W images revealed a two‐layered bladder wall, with a strongly enhancing inner layer, likely representing the mucosa and submucosa, and a hypointense outer layer that may represent the muscularis propria. This enhancement pattern is consistent with dynamic contrast‐enhanced (DCE) MRI in humans, where the highly vascular inner layers enhance earlier than the muscular layer [28]. Conversely, pre‐T1W images showed visible bladder wall layering in healthy dogs, with layers appearing relatively faint or slightly blurred compared to post‐T1W images. On T2W images, the normal bladder wall appeared as a single hypointense layer, consistent with human findings in which the detrusor muscle remains visible but the inner wall layers are obscured by the high signal intensity of urine [28]. In clinical cases, particularly Case 5, tumor invasion disrupted both bladder wall layers on post‐T1W images, suggesting extension into the muscular layer. Similarly, in Case 6, marked bladder wall thickening allowed the inner and outer layers to become more distinct on T2W images—unlike the normal bladder wall—suggesting that tumor‐associated structural changes, such as edema or infiltration, may enhance layer differentiation.

In human medicine, assessment of muscle invasion is a critical component of bladder cancer staging, as muscle‐invasive bladder cancer (MIBC) is associated with poorer prognosis and often necessitates radical cystectomy [28]. The Vesical Imaging‐Reporting and Data System (VI‐RADS) is used to predict muscle invasion by incorporating T2W, diffusion‐weighted imaging (DWI)/apparent diffusion coefficient (ADC) and DCE sequences [28]. These findings are clinically relevant in veterinary patients as well. Although studies on muscle‐invasive TCC are limited in veterinary medicine, high‐grade invasive TCC is known to be associated with a high risk of urinary tract obstruction, metastasis, and limited treatment options [13, 29, 30].

In addition to wall layer evaluation, T2W imaging enabled clear distinction between the mucosal layer and the urethral lumen by using hyperintense urine as a natural contrast medium. This property allowed non‐invasive assessment of luminal patency and tumor margins, suggesting that T2W imaging may serve as a complementary or alternative technique to retrograde urethrography, which is widely used and readily accessible in clinical practice. However, retrograde urethrography requires catheterization and contrast administration, which can lead to potential complications such as urethral trauma or contrast extravasation [10]. In Case 6 with a bladder tumor with prostatic invasion, disruption of the mucosal layer–lumen interface was observed in the ventral prostatic urethra, strongly indicating mucosal invasion. Similarly, in Case 4, the bladder tumor margins were clearly delineated against the hyperintense urine on T2W images, enhancing tumor conspicuity. The use of urine as a natural contrast medium in T2W imaging has been reported in human medicine, where T2W three‐dimensional (3D) MR urography provides superior urinary tract visualization compared to T1W 3D contrast medium enhanced MR urography, particularly in patients with renal impairment or contraindications to iodinated contrast agents [31]. These findings support the diagnostic value of T2W imaging not only for structural assessment but also for enhancing tumor detection and staging in lower urinary tract neoplasia.

Post‐T1W images showed superior conspicuity of the outer membranous urethra, likely due to its high sensitivity to differences in longitudinal relaxation times, which enhances contrast differentiation between fat and muscle [32, 33]. This effect may have been further influenced by variations in muscle composition and differential enhancement between the membranous urethra and adjacent tissue. The improved contrast resolution on post‐T1W images allowed clearer delineation of the membranous urethra from surrounding musculature, aiding in the assessment of tumor extent. In Cases 2 and 3, both prostatic tumors caused rectal compression; however, an intact hypointense rectal muscular layer on post‐T1W and T2W images suggested the absence of rectal wall invasion.

In healthy dogs, the bladder wall layer was visible on pre‐T1W images, although the distinction ranged from barely visible to slightly blurred. In contrast, these layers were nearly indistinguishable in all clinical cases, possibly due to the smaller body size of affected dogs compared to normal dogs. Smaller overall anatomical structures may have exceeded the spatial resolution limits of MRI, leading to partial volume effects. Additionally, small patients are more susceptible to motion artifacts, which may further degrade image quality. These findings demonstrate the importance of contrast enhancement for the accurate evaluation of bladder wall lesions and the added diagnostic value of post‐T1W imaging in dogs with urinary tract tumors. Furthermore, the determination of bladder muscular invasion or urethral wall involvement using MRI is clinically significant, as it directly influences treatment planning [2, 3, 4, 5, 34]. Evidence of bladder TCC with transmural invasion or urethral tumor may preclude curative surgical treatment such as partial cystectomy and prompt the use of palliative therapies such as radiation therapy, chemotherapy, or medical management. In this study, all dogs having the uroepithelial tumor underwent radiation therapy, and MRI was fused with CT for radiotherapy planning to allow precise tumor contouring. MRI enabled accurate assessment of tumor extension, identifying prostatic urethral invasion in some cases and exclusion of rectal wall invasion in others. This information contributed to more accurate radiation field planning, minimizing the risks of under‐ or overtreatment and associated adverse effects. CT provided optimal image quality with minimal artifacts, whereas both pre‐ and post‐ T1W were more susceptible to artifacts that impaired image evaluation. Motion artifacts arising from breathing, bowel movement, and bladder contractions frequently resulted in ghosts or smearing on MRI, particularly due to the longer acquisition time of T1W sequences [35, 36]. Additionally, susceptibility artifacts from air pockets trapped between the dogs and the flex coil, or from structures located outside the effective range of the flex coil, contributed to localized signal loss. For example, in Case 4, image evaluation was significantly compromised by such artifacts, making the bladder wall layers and tumor margin indistinct on both pre‐ and post‐T1W. Even CT imaging in this case showed blooming artifacts caused by intravesical contrast medium, which further impaired identification of tumor margins. In contrast, T2W delineated the tumor margins more clearly by using hyperintense urine as a natural contrast medium.

The use of an 8‐channel flex coil in MR urethrography was advantageous for conforming to the abdominal contour and improving signal uniformity across a wide FOV. To minimize signal drop‐off at the boundaries of the coil, the FOV was adjusted to specifically cover the region from the bladder neck to the distal membranous urethra in the transverse plane. Although image degradation was still noted when anatomical structures extended beyond the range of the coil, flex coils offered practical benefits for small animal imaging due to their adaptability to various body shapes and ability to provide a high signal‐to‐noise ratio (SNR) when closely fitted, demonstrated in humans [11, 24, 37, 38].

This study had certain limitations. First, only a small number of dogs were included to assess the normal lower urinary tract, and most of them were male. Further studies in a larger population with a balanced distribution of sex, as well as variation in breed and body sizes, are required to broaden anatomical understanding and to account for potential sex‐related differences in urethral or bladder wall imaging. Second, there was no standardized preparation protocol for lower urinary tract imaging. The absence of enemas resulted in retained fecal material in the rectum, which in some cases caused urethral compression and may have affected image interpretation. The urethral lumen was not distended using saline or gel, which may have reduced the conspicuity of the inner urethral layer‐lumen margin. Future studies with standardized bowel preparation and controlled urethral distension may help improve image quality and reproducibility. Third, BRAF mutation testing was not consistently performed in all clinical cases with suspected urothelial carcinoma, although diagnoses were based on imaging findings, cytologic evaluation, and clinical presentation. Finally, the number of dogs was small, and the statistical power may be insufficient. A post hoc power analysis using G*Power 3.1 indicated that at least 13 animals would be required to detect a medium to large effect size (*f* = 0.35) with *α* = 0.05 and power = 0.80. Further studies with larger study populations are necessary to enhance statistical power and confirm and generalize the present findings.

In conclusion, MR urethrography was a feasible and valuable modality for assessing the canine lower urinary tract. T2W sequences enabled anatomical layer visualization and enhanced lumen and tumor margin conspicuity using urine as a natural contrast agent, while post‐T1W sequences improved tissue boundary delineation and detection of tumor invasion. Although MRI is not routinely used due to cost and limited accessibility, it can offer diagnostic benefits, particularly in cases requiring detailed soft tissue evaluation. MR urethrography represents a non‐invasive alternative to retrograde urethrography, avoiding the need for catheterization or intraluminal contrast and enhancing its clinical applicability.

## Disclosure

Authors declare no off‐label use of antimicrobials.

## Ethics Statement

This study was conducted with the approval of the Institutional Animal Care and Use Committee (IACUC) of Seoul National University, and all animals were maintained according to the Guidelines for Animal Experiments of Seoul National University (SNU IACUC‐24010‐3). Informed owner consent was obtained for all client‐owned patients prior to inclusion in the study. Authors declare human ethics approval was not needed.

## Conflicts of Interest

The authors declare no conflicts of interest.
